# Distinct Differentiation Characteristics of Endothelium Determine Its Ability to Form Pseudo-Embryos in Tomato Ovules

**DOI:** 10.3390/ijms21010012

**Published:** 2019-12-18

**Authors:** Inna Chaban, Ekaterina Baranova, Neonila Kononenko, Marat Khaliluev, Elena Smirnova

**Affiliations:** 1 All-Russia Research Institute of Agricultural Biotechnology, Timiryazevskaya 42, Moscow 127550, Russia; inna_chaban@rambler.ru (I.C.); greenpro2007@rambler.ru (E.B.); nilava@mail.ru (N.K.); marat131084@rambler.ru (M.K.); 2 Moscow Timiryazev Agricultural Academy, Russian State Agrarian University, Timiryazevskaya 49, Moscow 127550, Russia; 3 Biology Faculty, Lomonosov Moscow State University, Leninskie Gory 1/12, Moscow 119234, Russia

**Keywords:** development, embryo sac, endosperm, endothelium, integument, ovule, pseudo-embryo, tomato, *Solanum lycopersicum*

## Abstract

The endothelium is an additional cell layer, differentiating from the inner epidermis of the ovule integument. In tomato (*Solanum lycopersicum* L.), after fertilization, the endothelium separates from integument and becomes an independent tissue developing next to the growing embryo sac. In the absence of fertilization, the endothelium may proliferate and form pseudo-embryo. However, the course of the reorganization of endothelium into pseudo-embryo in tomato ovules is poorly understood. We aimed to investigate specific features of endothelium differentiation and the role of the endothelium in the development of fertilized and unfertilized tomato ovules. The ovules of tomato plants (“YaLF” line), produced by vegetative growth plants of transgenic tomato line expressing the *ac* gene, encoding chitin-binding protein from *Amaranthus caudatus* L., were investigated using light and transmission electron microscopy. We showed that in the fertilized ovule of normally developing fruit and in the unfertilized ovule of parthenocarpic fruit, separation of the endothelium from integument occurs via programmed death of cells of the integumental parenchyma, adjacent to the endothelium. Endothelial cells in normally developing ovules change their structural and functional specialization from meristematic to secretory and back to meristematic, and proliferate until seeds fully mature. The secretory activity of the endothelium is necessary for the lysis of dying cells of the integument and provides the space for the growth of the new sporophyte. However, in ovules of parthenocarpic fruits, pseudo-embryo cells do not change their structural and functional organization and remain meristematic, no zone of lysis is formed, and pseudo-embryo cells undergo programmed cell death. Our data shows the key role of the endothelium as a protective and secretory tissue, needed for the normal development of ovules.

## 1. Introduction

One of the basic features of the ovule development in tomato is the formation of new tissue—the endothelium—differentiating from the inner layer of singular integument, and assembling an extra boundary tissue around the embryo sac. If fertilization does not occur, the endothelium may separate, grow, and proliferate independently of other tissues. This eventually leads to the disruption of the embryo sac and development of a pseudo-embryo—the additional tissue—which substitutes the embryo. This unique feature of the endothelium in plants from the *Solanaceae* family was observed during the development of ovules in hybrid plants [1,2,3]. According to these studies, rapidly proliferating endothelium may induce death not only of embryo sacs but also developing embryos. Pseudo-embryos were often detected in the ovaries of parthenocarpic tomato fruits [4,5,6,7], but it is still unclear how the proliferating endothelium contributes to pseudo-embryo formation.

In our previous study, we presented morphological, anatomical, and embryological data on the abnormal morphogenesis of generative organs in subpopulations of tomato plants after genetic transformation [8]. We did not find any anomalies during female gametophyte development, but observed significant changes during male gametophyte formation, leading to pollen sterility. Despite the failed fertilization, the ovule differentiation continued due to the proliferation of endothelial cells, which formed pseudo-embryo instead of a degenerating embryo sac. Apparently, the process of imitating embryogenesis stimulates the development of the ovary, which then forms a parthenocarpic fruit.

The objective of this study was to investigate structural characteristics of the endothelium differentiation during the normal development of fertilized tomato ovaries and to describe the specific features of the endothelium differentiation into the pseudo-embryonic tissue in unfertilized ovaries of transgenic tomato expressing the *ac* gene, encoding a chitin-binding protein. This study may contribute to the general understanding of the functional role of the endothelium during tomato seed development.

## 2. Results

**Figure 1:**
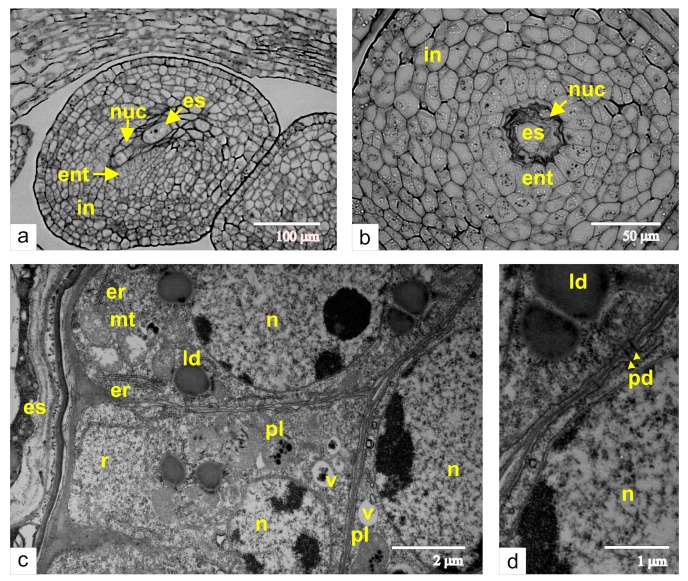
Early stages of the endothelium formation: (**a**) a longitudinal section of the ovule at the stage of the haploid megaspore; (**b**) a cross-section of the same ovule through the region of dead cells of the nucellus; (**c**) an ultrastructural image of the endothelium from the same stage of growth; (**d**) plasmodesmata between endothelial cells and cells of integumental parenchyma. Abbreviations: ent, endothelium; es, embryo sac; in, integument; ld, lipid droplet; mt, mitochondria; nuc, nucellus; n, nucleus; pd, plasmodesmata; pl, plastid; v, vacuole.

As a primary step of embryo sac and endothelium differentiation, we analyzed the stage of haploid megaspore detachment. At the time of the embryo sac formation, the single-layered nucellus is lysed, and the mature embryo sac interacts with the inner epidermal layer of the integument. The cells of this integument layer then differentiate into the endothelium, also termed as integumental tapetum (Figure 1). The longitudinal section of the ovule in the stage of the unicellular embryo sac (Figure 1a) shows the degenerating nucellus and disrupting megaspores. The integument is composed of seven to ten cell layers. The cross-section of the ovule at the same stage of development demonstrates that the endothelium cells expand perpendicular to the axis of the embryo sac (Figure 1b). The endothelium cells show typical ultrastructural characteristics for meristematic tissues: multiple free ribosomes, random cisternae of rough endoplasmic reticulum, lipid droplets, and small vacuoles (Figure 1c). Additionally, the endothelial cells are connected via plasmodesmata with the cells of integumental parenchyma (Figure 1d).

The subsequent development of the embryo sac leads to the formation of the female gametophyte. As we showed in our previous study, this process is not altered in tomato plants with an infertile phenotype [8,9].

**Figure 2:**
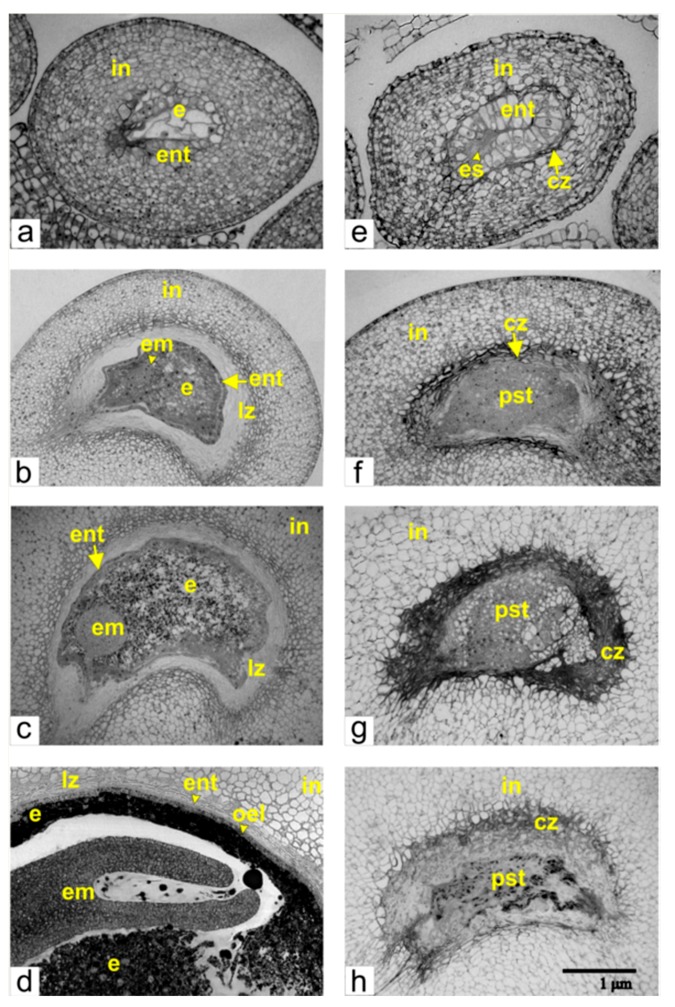
Developing ovules from normal (**a**–**d**) and transgenic (**e**–**h**) tomato during corresponding phases of growth: (**a**) a phase of a two cellular pro-embryo and eight-ten cellular endosperm; (**b**) early multicellular embryo; (**c**) globular-shaped embryo; (**d**) torpedo-shaped embryo; (**e**) the beginning of the endothelium proliferation in unfertilized ovule and degeneration of the embryo sac; (**f**) the pseudo-embryo at the last stage of proliferation; (**g**) the beginning of the pseudo-embryo degeneration; (**h**) a fully degraded pseudo-embryo. Abbreviations: cz, compression zone; em, embryo; e, endosperm; ent, endothelium; es, embryo sac; in, integument; lz, lysis zone; oel, outer endosperm layer; pst, pseudo-embryonic tissue.

Then we studied the development of ovules after fertilization in non-transformed (normal) tomato and compared it with the development of the unfertilized abnormal ovules from transgenic tomatoes. We matched all phases according to the stages of the ovule growth (Figure 2).

### 2.1. Development of Ovules after Fertilization in Normal Tomato

The development of the embryo and endosperm in the embryo sac is linked to the growth of somatic tissues of the ovule—the endothelium and the integument. The longitudinal sections of the ovules during consecutive stages of development showed a two-celled pro-embryo, an early multicellular embryo, a globular-shaped embryo, and a torpedo-shaped embryo (Figure 2).

After fertilization, the endosperm and the endothelium simultaneously began to proliferate. During this phase, the endosperm was composed of eight to ten large and highly vacuolated cells (Figure 2a) and positioned close to the endothelium, whereas degrading and cells of integumental parenchyma appeared between the endothelium and the integument.

The analogous course of development is observed during the subsequent early stages of pro-embryo proliferation when the number of endosperm cells increases. During the formation of the multicellular embryo (Figure 2b), the endothelium segregated from the endosperm, and between the endothelium and the integument, the lysis zone was formed from degrading cells of the integumental parenchyma.

At the stage of the globular-shaped embryo (Figure 2c), the endosperm differentiated into peripheral and central zones. Cells from the central zone accumulated large amounts of storage components, predominantly starch and lipid inclusions. The peripheral zone of the endosperm is composed of one or two cell layers, and these cells have very few storage inclusions. At the stage of the torpedo-shaped embryo (Figure 2d), the embryo and the endosperm (except its peripheral zone) accumulated a substantial amount of storage inclusions. The endothelium adjoined the endosperm, and the lysis zone was thinner than in the preceding phases of the development.

### 2.2. Development of Unfertilized Ovules in Transgenic Tomato

**Figure 3:**
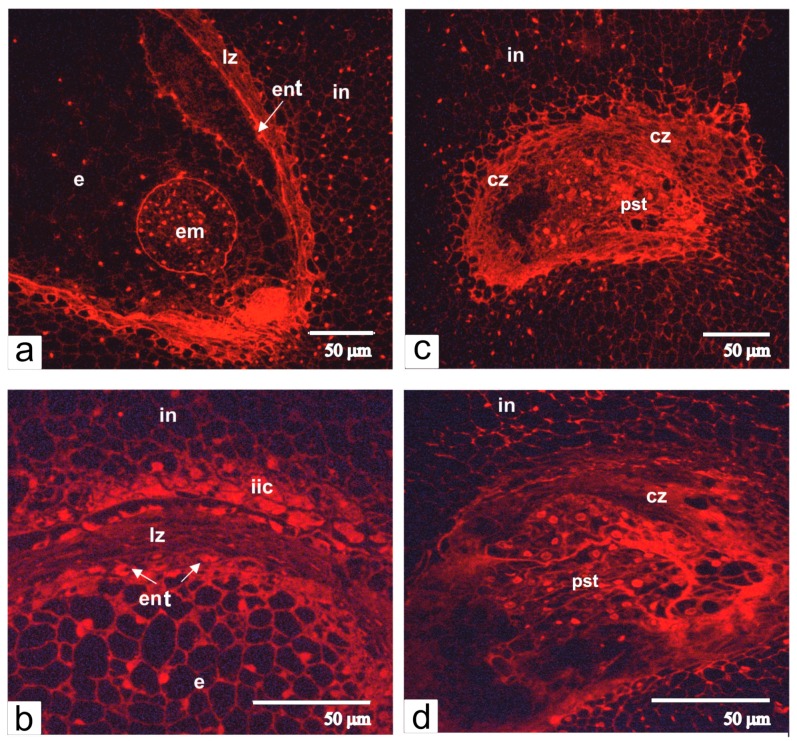
Semithin-sections of ovules from normal (**a**,**b**) and transgenic (**c**,**d**) tomato are stained with propidium iodide. Abbreviations: in, integument, cz, compression zone, ent, endothelium; em, embryo; e, endosperm; lz, lysis zone; pst, pseudo-embryonic tissue; iic, inner integument cells.

In the ovules of transgenic tomato with altered phenotype, fertilization did not occur, and therefore the embryo and the endosperm were not formed. However, the differentiation of integument and the proliferation of endothelium were not halted (Figure 2e,h), dividing endothelium cells gradually filled in the area the embryo sac and generated its compression and degradation (Figure 2e). Cells of growing endothelium acquired an elongated shape and assembled a single-layered tissue, which at some places folded and produced invaginations. This resulted in the clustering of cells and the formation of a pseudo-embryo, a special tissue that replaced the undeveloped embryo sac (Figure 2f). Staining with propidium iodine showed that nuclei of pseudo-embryos were much bigger than nuclei of integumental parenchyma cells (Figure 3).

As the ovule continued to grow, the size of pseudo-embryo increased, and the area of dead integumental cells, which encircled the pseudo-embryo, also expanded (Figure 2g). In contrast to the degraded cells of integumental parenchyma during the development of fertilized ovules, the integumental cells in unfertilized ovules of transgenic tomato were not lyzed and remained as rigid layers of dead cells. These layers produced a spatial restriction, limit cellular growth, and induced the degeneration of the pseudo-embryo. At this stage of differentiation, the pseudo-embryo was composed of larger vacuolated cells and smaller cells, located in its central part (Figure 2g). Gradually the pseudo-embryo transformed into an amorphous aggregate (Figure 2h), surrounded by live and non-differentiated integumental cells. Accordingly, the maturing parthenocarpic tomato fruit contained only small and undeveloped ovules.

During unfertilized/abnormal ovule development in transgenic plants, the thickness and the number of integument cell layers (20–24) did not change, and remained the same as in the ovules of non-transformed tomatoes at the stage of the globular-shaped embryo.

We then used transmission electron microscopy for detailed structural analysis of the development and differentiation of all tissues at the most critical phases of the ovule growth.

### 2.3. Normal Tomato, the Phase of Two-Cellular Proembryo

**Figure 4:**
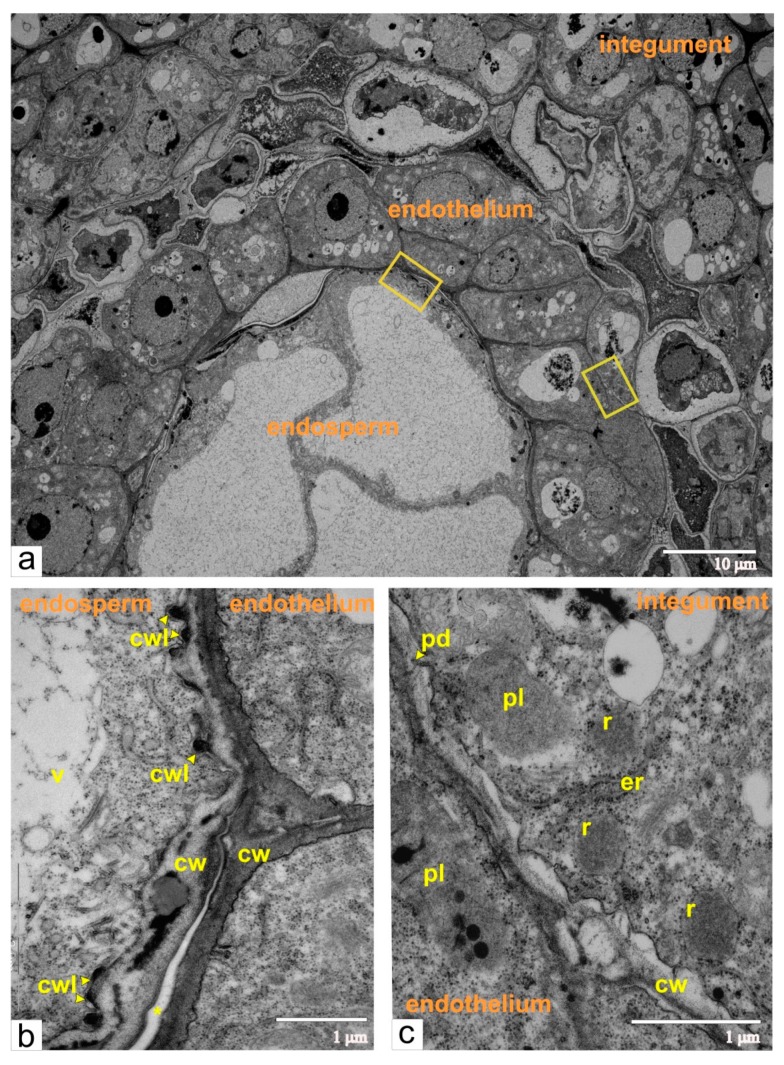
Ultrastructural images of the cross-sectioned ovule from normal tomato at the double-cellular pro-embryo, and eight to ten cellular stage endosperm: (**a**) a panoramic image of the area with endothelium, endosperm, and integument, yellow rectangles indicate the fragments shown on (**b**,**c**); (**b**) enlarged zone of the contacts between the endothelium and the endosperm; (**c**) enlarged zone of the contact between the endothelium and the integument; integument cells have ricinosomes as morphological markers of programmed cell death (PCD). Abbreviations: cw, cell wall; er, endoplasmic reticulum; cwl, cell wall labyrinth; pd, plasmodesmata; r, ricinosome; v, vacuole; pl, plastid.

At the stage of the two-cellular pro-embryo, the endosperm contained eight to ten large cells. Each cell had a central vacuole and thin layer of cytoplasm with a structural organization typical for meristematic cells (Figure 4a). Outer walls of endosperm cells firmly adjoined walls of endothelial cells, and occasionally the remnants of dead nucellus cells were seen between endosperm and endothelium. We also observed numerous ingrowths of endosperm walls forming labyrinths toward the cytoplasm, which substantially increased the surface of the plasma membrane (Figure 4b).

At the stage of the early embryo sac development, the endothelial cells had a typical ultrastructural organization of meristematic cells (many free ribosomes, random cisternae of endoplasmic reticulum, plastids with a few lamellae and globular inclusions, juvenile mitochondria, and numerous vacuoles with flake-like inclusions). The degenerating cells of the integumental parenchyma surrounded the endothelium. The cytoplasm of these cells segregated from cell walls and contained ricinosomes, but lacked plasmodesmata (Figure 4c), which cumulatively indicated the progression of PCD (programmed cell death) [10,11,12].

### 2.4. Normal Tomato, Phase of 8-Cellular Proembryo

**Figure 5:**
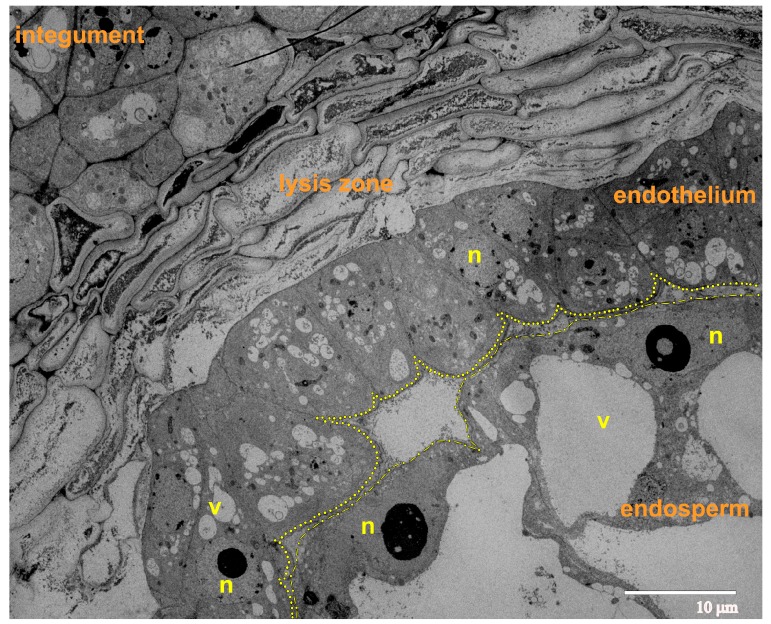
Ultrastructural image of the ovule (longitudinal section) during the phase of 8-cellular proembryo; the yellow dots delineate the gap between the endothelium and the endosperm. Abbreviations: n, nucleus; v, vacuole.

At the 8-cellular pro-embryo stage of development, we observed progressing growth and structural reorganization of the endosperm cells. The overgrowth of the endothelium occurs mostly due to the proliferation of the cells rather than the extension of the cells, along with the growing endosperm (Figure 5). The endothelium cells showed a flattened shape, and in some areas, were organized in two layers. The endothelium was not yet separated from the endosperm and was adjacent to it. This prevents the stretching of endothelium cells along the surface of the growing endosperm. On the opposite side, the endothelium was separated from the integument by a considerably increased lysis zone, formed by several layers of partially or almost completely lysed cells of the integumental parenchyma.

### 2.5. Normal Tomato, Phase of Early Multicellular Embryo

**Figure 6:**
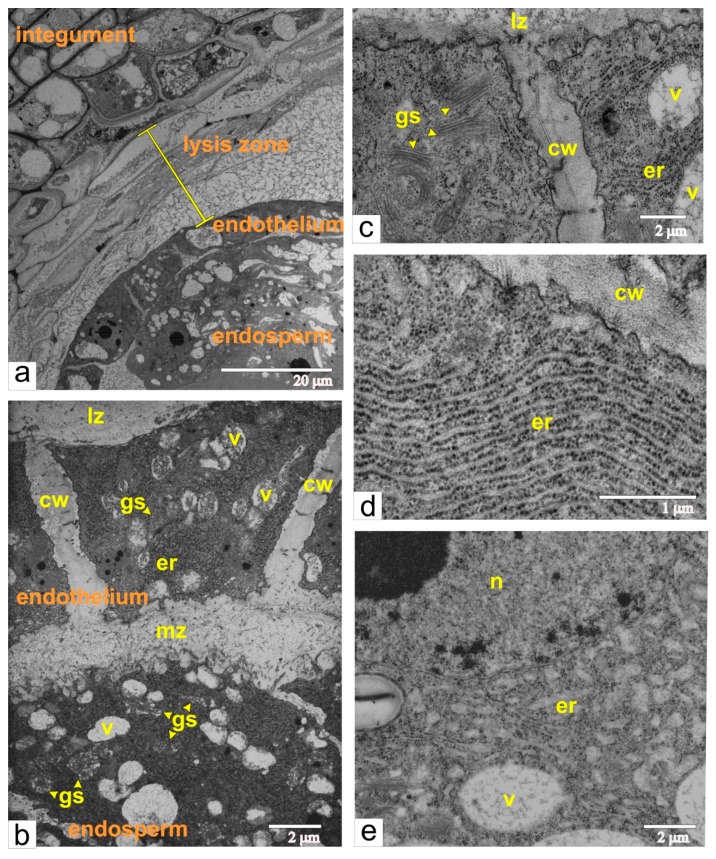
Ultrastructural images of the ovule at the phase of multicellular embryo and during separation of the endothelium form the endosperm and formation of the mucilage zone: (**a**) a panoramic view; (**b**) accumulation of the mucilage between the endothelium and the endosperm; (**c**) endothelium cells localized next to the lysis zone; (**d**) ER arranged as ergastoplasma in the endothelial cell; (**e**) ER cisternae in the endosperm cell. Abbreviations: er, endoplasmic reticulum; lz, lysis zone; mz, mucilage zone; n, nucleus; s, starch; v, vacuole; gs, Golgi stack; cw, cell wall.

Beginning with the formation of the multicellular body of the embryo, the structural reorganization of the endothelium and the endosperm cells took place and proceeded concomitantly with the splitting and complete segregation of these tissues (Figure 6a,b). At this phase, the endothelium and the endosperm cells have similar structural characteristics, such as very dense cytoplasm, numerous cisternae of the endoplasmic reticulum (ER) and Golgi stacks, vacuoles with fibrous or flake-like inclusions. However, the successive changes of the ER in each tissue occur in different ways. Endothelial cells acquired elongated cisternae of endoplasmic reticulum, which were often regularly packed and assemble ergastoplasma (Figure 6d), whereas endosperm cells had variably shaped and short cisternae (Figure 6e). The space between the endothelium and endosperm was filled with granular or/and fibrous material (Figure 6b). The plasma membranes at the inner cell walls of both tissues bordering each other had a sinuate contour, most evident in the endosperm cells. According to many authors, such ultrastructural composition is common for cells producing polysaccharide mucilage [13,14,15,16,17,18].

Apparently, mucilage was also incorporated into radial walls of the endothelial cells, and as a result, cells were separated from each other by broad intercellular space. These walls may have provided spatial integration between the mucilage and lysis zones (Figure 6b,c). The outer walls of endothelial cells that were in contact with the lysis zone were quite thin and lacked cuticles (Figure 6c).

### 2.6. Normal Tomato, Phase of Globular-Shaped Embryo

**Figure 7:**
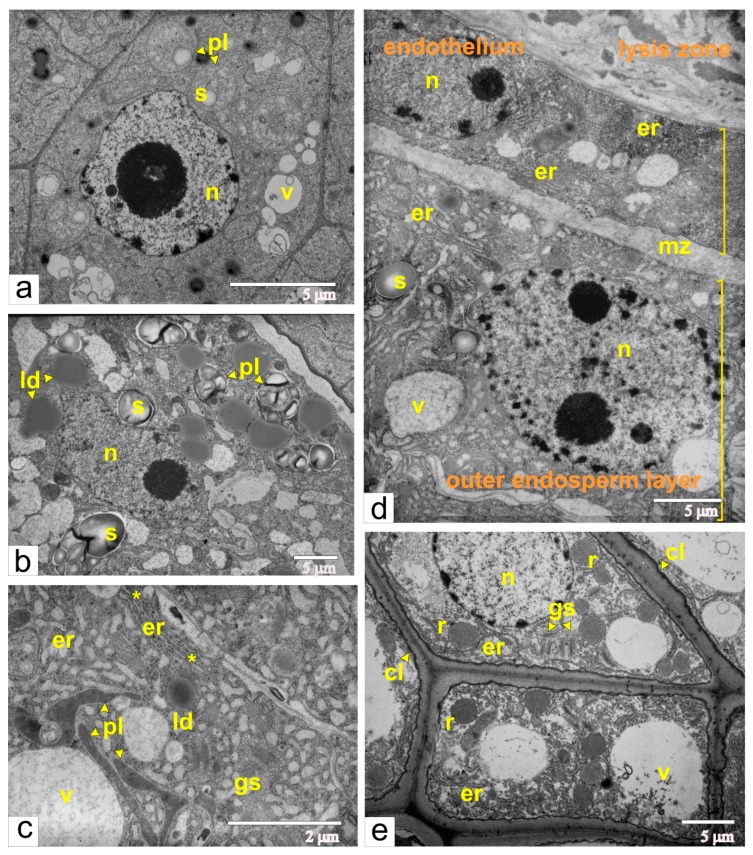
Ultrastructural images of cells from different tissues of the ovule during the phase of globular-shaped embryo: (**a**) the embryo cell; (**b**) the cell from the central area of the endosperm; (**c**) the cell from the peripheral area of the endosperm; aster shows the dilations of the ER termini; (**d**) a panoramic view of the area of the mucilage zone, the endothelium and cells from the peripheral part of the endosperm; (**e**) ricinosomes in the cells of the inner integument. Abbreviations: er, endoplasmic reticulum; gs Golgi stack; cl, callose; ld, lipid droplet; mz, mucilage zone; n, nucleus; pl, plastid; r, ricinosome; s, starch; v, vacuole.

The globular-shaped embryo is a crucial stage during ovule development. At this stage, cells of the developing embryo were characterized by a large number of free ribosomes, very few cisternae of endoplasmic reticulum, small vacuoles, juvenile mitochondria, plastids with prolamellar bodies and small starch grains (Figure 7a). As shown previously, this ultrastructural organization is typical for meristematic cells. Additionally, endothelial cells become differentiated and metabolically active, and endosperm differentiates into central and peripheral zones.

The central zone included cells with few cisternae of endoplasmic reticulum, numerous lipid droplets, and plastids with large starch granules (Figure 7b). The same ultrastructural characteristics are common for storage tissues. On the contrary, peripheral endosperm cells had an extensive network of rough endoplasmic reticulum, organized as flat cisternae and tubular extensions, and scarce tubules of smooth ER (Figure 7c). Additionally, the termini of cisternae were often dilated. The extensive network of the endoplasmic reticulum, in addition to the numerous Golgi stacks and vacuoles with fibrous or granular inclusions, cumulatively indicated that peripheral endosperm cells are involved in biosynthetic processes.

At this stage, the endothelial cells also had an extensive network of the rough endoplasmic reticulum. Flattened cisternae often stack together and assemble ergastoplasma, and vacuoles are filled with flake-like or fibrous material. This cumulatively indicates that endothelial cells are also synthetically active. Concomitantly, the space between endosperm and endothelium narrowed (Figure 7d), and the contour of the plasma membrane became less sinuate. Due to the accumulation of mucilage, the radial walls of the endothelial cells were still enlarged. As a result, the endothelium was covered on all sides by mucilage.

**Figure 8:**
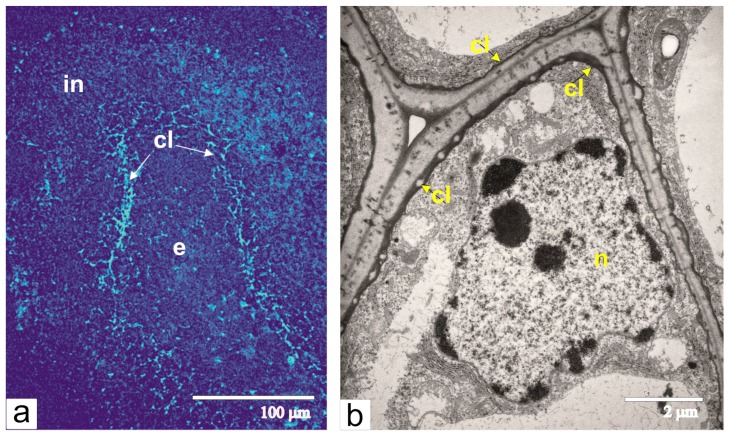
Callose in the integument cells adjacent to the lysis zone: (**a**) detection of callose fluorescence with aniline blue, arrows show the sites of callose (cl) deposits; (**b**) an ultrastructural image of the integument cell with the layer of callose deposit between the cell wall and plasma membrane. Abbreviations**:** cl, callose; e, endosperm; in, integument; n, nucleus.

At this time, the integument acquires its maximal thickness, and most of its parenchymal cells are extremely vacuolated. Unlike cells from the main part of the multilayered integument, cells from the inner layers of the integumental parenchyma, near the lysis zone, are visibly larger. Characteristic features of these cells are thickened walls and electron-dense deposits of callose between the plasma membrane and cell wall, which was confirmed by callose luminescence after staining with aniline blue (Figure 8a,b).

Moreover, cells with callose deposits contained numerous ricinosomes, which are often budded from the ER cisternae (Figure 7e). As shown previously (Figure 4c), ricinosomes were formed in the cells of inner integumental layers after fertilization and were detected during each phase of the ovule development.

### 2.7. Normal Tomato, Phase of Torpedo-Shaped Embryo

**Figure 9:**
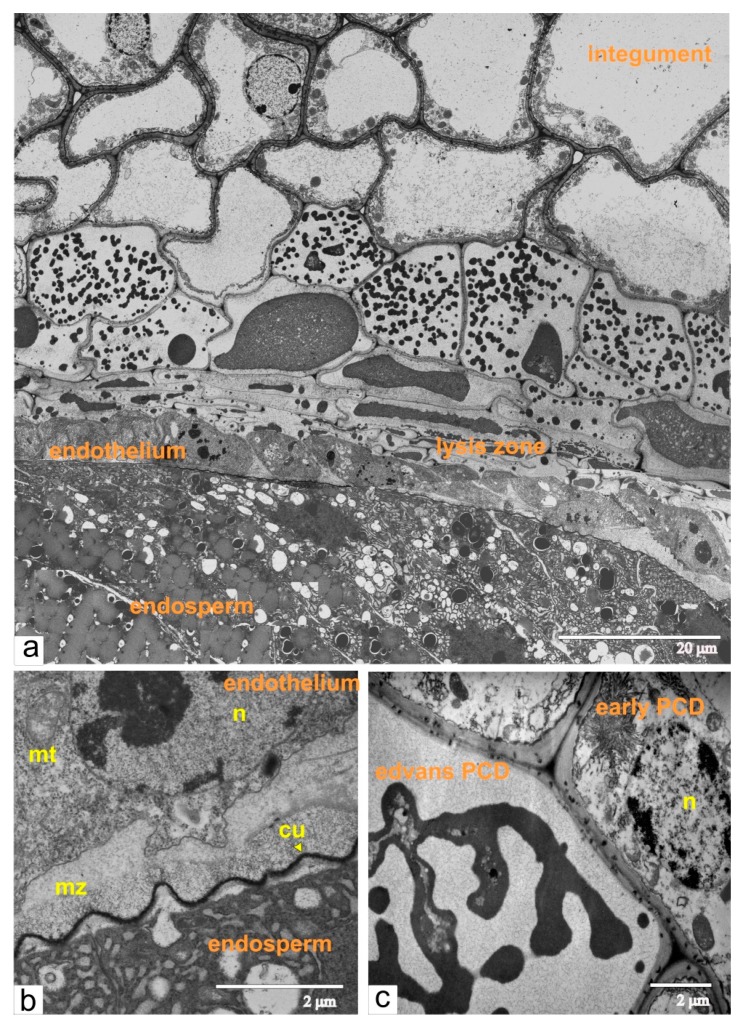
Ultrastructural organization of cells from the ovule during the phase of torpedo-shaped embryo: (**a**) a panoramic view shows the area of endothelium, endosperm, lysis zone and dying integument cells; (**b**) the endothelium cell located next to the endosperm; (**c**) the integument cell in advanced stage of PCD (shrinking protoplast) and neighboring cells with early signs of PCD. Abbreviations: cu, cuticle; mt, mitochondria; mz, mucilage zone; n, nucleus.

At the torpedo-shaped embryo stage, endothelial cells yet again acquired the ultrastructural organization of undifferentiated meristematic cells (Figure 9a). They also showed a slightly elongated shape and were disjoined due to amorphous radial walls. The cells of endosperm and embryo have dense cytoplasm, an extensive network of endoplasmic reticulum, numerous vacuoles and a lot of storage material. Outer endosperm walls, contacting the endothelium, were covered with a thin layer of sinuate cuticle (Figure 9b). The mucilage zone between the endothelium and the endosperm was clearly partitioned into areas, which can be attributed to each of these tissues. The outer region of the endothelium directly contacted the layer of dead cells of integumental parenchyma, which were not yet completely lyzed, as evidenced by the preserved cell walls and protoplast remnants in the form of a dense homogeneous mass (Figure 9c).

The overall process of the cell death in the integument could be followed on the fragment of the longitudinal section of the ovule (Figure 9a). Integumental cells, located close to the lysis zone, had numerous ricinosomes and vacuoles. Subsequently, the protoplast shrank and was fragmented into vesicles (Figure 9a–c), which then fused into an amorphic mass. Endothelial cells maintain their structural organization until the complete maturation of the seeds. Sequential development of the ovules and ovaries produce ordinary tomato fruit with fully differentiated seeds.

### 2.8. Transgenic Tomato, Beginning of Endothelium Proliferation in an Unfertilized Ovule

**Figure 10:**
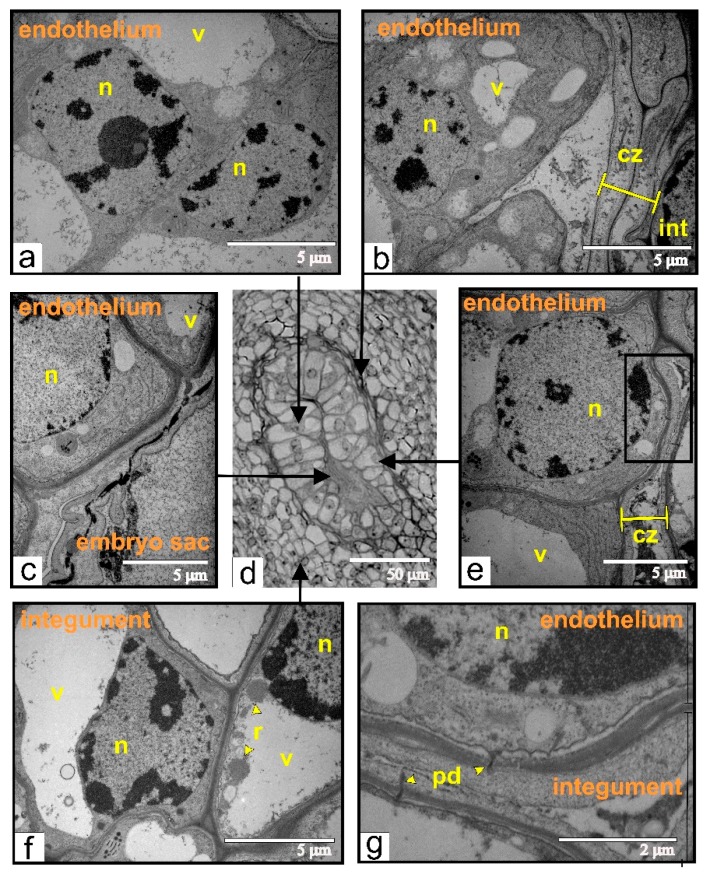
Ultrastructural organization of cells during early stages of endothelium proliferation (**a**–**c**,**e**–**g**) taken from different zones (indicated by black arrows) of the endothelium from the ovule (**d**) of transgenic tomato: (**a**) the cell from the middle part of the pseudo-embryo; (**b**) endothelial cells, separated from the dead and compressed integument; (**c**) endothelial cells localized next to the dead embryo sac; (**d**) semi-thin section of the ovule used for ultrastructural analysis; (**e**) the endothelial cell connected via plasmodesmata with compressing and degenerating integumental cells; the black rectangle indicates the magnified area shown on “g”; (**f**) the integumental cells with ricinosomes; (**g**) the area shown in black rectangle on (**e**) and rotated 90° clockwise. Abbreviations: cz, compression zone; int, integument; n, nucleus; pd, plasmodesmata; r, ricinosome; v, vacuole.

At this phase of growth, pseudo-embryo cells showed an ultrastructural organization, which is typical for meristematic cells (Figure 10a,b). Dead cells formed one or two layers of integumental parenchyma, segregating the pseudo-embryo from the integument (Figure 10b). Adjacent to the dead zone, integument cells contained ricinosomes (Figure 10f). The endothelium was either fully (Figure 10b) or partially (Figure 10e,g) detached from the integument. However, the walls of endothelium cells were still connected via plasmodesmata with disintegrating cells of integumental parenchyma.

### 2.9. Transgenic Tomato, Phase of Maximal Size of Pseudo-Embryo in Unfertilized Ovule

**Figure 11:**
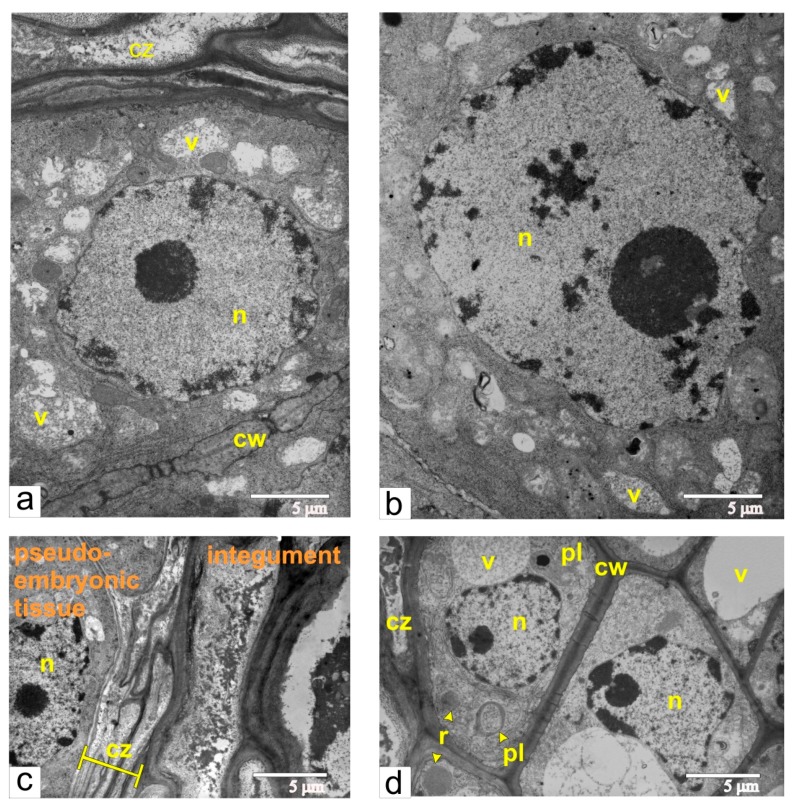
Pseudo-embryo during the final stage of active proliferation: (**a**) the area adjacent to the compression zone, composed of dead integumental cell; (**b**) the cell with large polyploid nuclei, from central part of pseudo-embryo; (**c**) compression zone next to the pseudo-embryo cell; (**d**) integument cells next to compression zone. Abbreviations: cw, cell wall; cz, compression zone; n, nucleus; mt, mitochondria; pl, plastid; r, ricinosome; v, vacuole.

At the stage of maximal size for the pseudo-embryo in unfertilized ovules, the characteristic features of the pseudo-embryo cell were rare cisternae of ER and numerous small vacuoles, filled with flake-like inclusions (Figure 11a).

**Figure 12:**
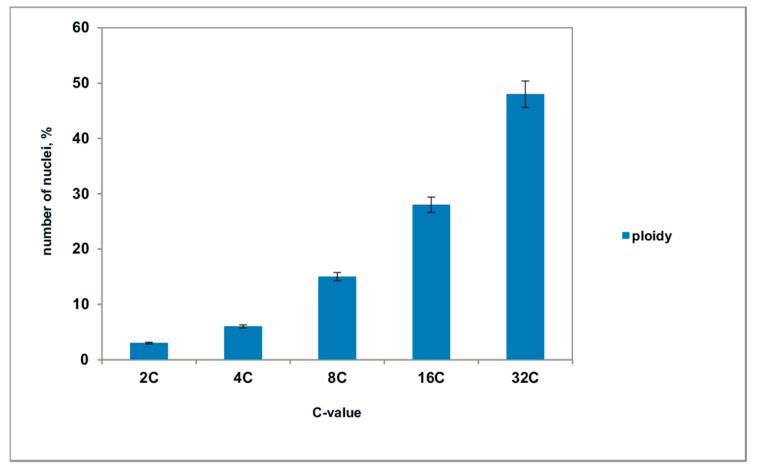
The level of ploidy in the nuclei of the pseudo-embryo cells, isolated from the ovules of transgenic tomato. The mean values and their standard deviations are shown according to Student’s criterion, *p* < 0.05.

Many cells had enlarged nuclei (Figure 11b), and cytophotometric investigation confirmed that the ploidy level of the nuclei in these cells varied from 2 °C to 32 °C (Figure 12). As shown in Figure 11c, the area of dying integumental cells compressed and notably expanded. Integumental cells, which were located nearby, had many ricinosomes (Figure 11d) and thickened cell walls but did not have callose deposits.

### 2.10. Transgenic Tomato, Beginning of Pseudo-Embryo Degradation in Unfertilized Ovule

**Figure 13:**
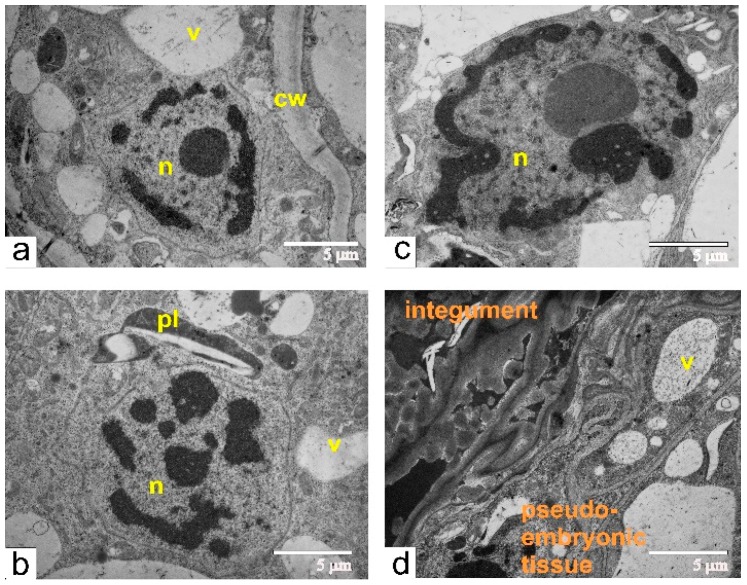
Ultrastructural organization of cells during the early stage of pseudo-embryo degeneration: (**a**–**c**) cells during various stages of PCD; (**d**) compressed cells of integument and peripheral layers of pseudo-embryo. Abbreviations: cw, cell wall; pl, plastid; r, ricinosome; n, nucleus; v, vacuole.

Once the pseudo-embryo reaches the maximal growth phase (corresponding to the stage of the globular shaped embryo during normal ovule development), its cells undergo degradation. The degradation is first detected in cells of the peripheral part of pseudo-embryo. These cells are localized next to the compression zone, which is composed of dead cells of integumental parenchyma. Then the degradation affects younger cells from the central part of the pseudo-embryo. We noticed that as cells from the central part looked undamaged, the outer layers had typical features of PCD progression such as numerous ricinosomes (Figure 13a), dissociation of chromatin from the nuclear envelope, aggregation of chromatin into dense clusters, segregation of the nucleoli (Figure 13b,c), fragmentation of endoplasmic reticulum, swelling of mitochondria, followed by the dehydration and shrinking of the protoplast. Ultimately, degenerating cells of the pseudo-embryo were incorporated into and increased the thickness of the zone of dead cells (Figure 13d). At this moment, the development of ovules that had reached a particular size due to the differentiation of pseudo-embryonic tissue stopped. Nonetheless, the development of the ovary was not terminated and resulted in the formation of parthenocarpic fruit.

## 3. Discussion

Tomatoes are generally used as model plants for fundamental and applied research, including studies of the growth and development of vegetative and generative organs. Such attention may be rationalized by the unique combination of biological features of tomato plants, such as a shortened life cycle, self-pollination, fast reproductive rate, and high activity of meristematic tissues [19]. The meristem activity is manifested in the formation of extra vegetative and generative shoots, often leading to the development of deformed or/and parthenocarpic fruit. In general, parthenocarpy is commonly observed among tomato hybrids and in altered growth conditions [20]. Deformed and parthenocarpic tomato fruit are also observed after genetic transformation. For instance, among primary transformants, we found plants with an infertile phenotype developed parthenocarpic fruit [9].

Our previous study showed that loss of male gametophyte development and formation of sterile pollen is the reason for failed fertilization in the ovules of transgenic tomatoes with an infertile phenotype [8]. We suggest that such pollen contains hormones, which may induce the development of pseudo-embryo, followed by the formation of parthenocarpic fruit. It is well-known that the experimental application of plant hormones on the stigma of tomato plants may induce parthenocarpy. It has been demonstrated that the formation of parthenocarpic fruit directly correlates with the development of pseudo-embryos inside underdeveloped ovules [7,21,22]. Also, the pollination may induce signal transduction and then gene expression cascade resulting in the alteration of synthesis and response to phytohormones [23]. However, it is still unknown what type of tissue is involved in the initiation of pseudo-embryo development.

One of the distinctive features of tomato ovule development is the formation of a new tissue, the endothelium, which differentiates from the inner layer of a single integument and builds an additional boundary layer around the embryo sac. The endothelium (or integumental tapetum) is identified among the members of 65 families, mostly dicotyledones, with tenuinucellate and unitegmal ovules. The differentiation of endothelium, specific structural features, stages of development, and functional roles were discussed by Kapil and Tiwari [24]. It has been suggested that the main function of the endothelium is to provide a supply of nutrients for the embryo sac, yet another hypothesis stated that endothelium might be a barrier and protective tissue between the integument and the embryo sac [25]. Additionally, the endothelium is considered a secretory tissue, which produces enzymes for lysis of integumental parenchyma cells, located next to the endothelium [24,26]. Available data show that the structure and functions of the endothelium largely rely upon its lifetime during the ovule ontogenesis, which in turn, depends on specific features of the embryo and endosperm development. The embryo and endosperm formation and development vary in plants of different systematic groups, and it seems that endothelium may have multiple functions depending on the specificity of embryo and endosperm development.

Currently, it is known that endothelium only forms pseudo-embryonic tissue in the ovules of parthenocarpic fruit in tomatoes. Given the great attention to various aspects of the development of parthenocarpy and unequivocal interest to tomatoes as a model object for various fields of research [19,27], we aimed to investigate the consecutive stages of the formation and growth of endothelium and surrounding tissues during the development of tomato ovules and to analyze the structural changes of the endothelium during formation of pseudo-embryo in the ovules of parthenocarpic fruit of transgenic tomatoes, which we used as model plants in our previous study [8].

### 3.1. First Stages of Ovule Development

Our study showed that after fertilization, the first stage of the ovule development depends on the beginning of two simultaneous events: the development of the embryo and endosperm, and the differentiation of integument. The differentiation of integument was manifested in two synchronous processes: (1) the proliferation of cells in the outer integumental layer; and (2) the degradation of cells from the inner integumental layer, adjacent to the embryo sac. The latter provided better conditions for unrestricted growth and expansion of the new sporophyte. We believed that the terminal differentiation of the integument leads to the execution of PCD in tomato ovules. This was confirmed by the presence of ricinosomes in the cells of inner integumental layers during the earliest stages of ovule development. Ricinosomes are formed from the terminal extensions of the ER and established as morphological markers of PCD in many plants [28,29,30,31,32]. Remarkably, ricinosomes appear long before any other changes typical for PCD are detected [7,10,12].

### 3.2. Dissociation of Endothelium from Integument

Our results showed that the dissociation of the endothelium from the integument occurs due to the destruction and death of cells of the integumental parenchyma, adjoining the endothelium. At first, both tissues lose plasmodesmata, and then a layer of degraded cells completely separates the endothelium from the rest of the integument. Consequently, the endothelium transforms into a new, independent, and monolayered tissue. Its main function is to protect the embryo and the endosperm from dying cells of the integumental parenchyma. In our previous study, we showed that the dissociation of the endothelium from the integument after fertilization with sterile pollen in the ovules of transgenic tomato follows the same pathway [8]. We believe that in spite of unsuccessful fertilization, phytohormones of sterile pollen induce PCD in integument cells. Dying by PCD parenchymal cells eventually separate the endothelium from the integument. Since the connection with a non-developing embryo sac is also lost, the endothelium begins to proliferate as meristematic tissue and assembles a pseudo-embryo.

### 3.3. Interconnection between Endothelium and Endosperm

During the early stages of the ovule development, cell walls of the endothelium are firmly attached to the embryo sac. The inner cell walls of the endothelium contact the outer cells walls of the developing endosperm. The presence of labyrinths on these endosperm walls implies that nutrients are transported from the endothelium to the embryo sac via the apoplast from the lysis zone. Usually, wall outgrowths are detected in transfer cells, which are often formed between adjacent tissues. Outgrowing cell walls of the developing endosperm facilitate the absorption of metabolites from the ovule tissues to the embryo sac [29].

### 3.4. Detachment of Endothelium from Endosperm

The beginning of the development of the globular-shaped embryo correlates with specific ultrastructural changes in endothelium and neighboring endosperm cells, such as an expansion of the ER network and Golgi stacks. These changes are typical for cells with activated biosynthetic processes. At the same time, the endothelium forms a broad space filled with polysaccharide (pectin) mucilage and consequently detaches from the integument. The structural characteristics of the Golgi complex in endothelium and integument cells indicate that both tissues contribute to the formation of the mucilage zone. The mucilage facilitates the independent growth of the endothelium, which occurs alongside the growth of the endosperm. However, the endothelium and endosperm seem to be functionally connected because we did not detect any cuticle-like barriers between them. This course of endothelium differentiation was not observed in other members of the *Solanaceae* family [33,34], but reported in ovules of *Impatiens walleriana* [26].

By the time of the late globular-shaped embryo, the endosperm is finally differentiated into the central (storage) and peripheral (secretory) zones, which, apparently, is a unique feature of the endosperm in tomato ovule. It has been suggested that endothelium cells synthesize lytic enzymes, which are active during lysis of dying cells of integumental parenchyma [24,25,26], and lyzed products are utilized later for the nutrition of the growing embryo sac [15,17,18].

Additionally, during the differentiation of the integument, we observed callose deposits at the inner surface of the cell walls in several layers of integumental parenchyma adjacent to the lysis zone. The reinforcement of cell walls with callose is one of the main reactions of plant tissues in response to biotic stress [35,36,37]. We suggest that cells with callose deposits protect the other integumental cells from the aggressive influence of the lysis zone. This may be confirmed by the observation that in the abnormally developing ovules of transgenic tomato, in which the lysis zone around the pseudo-embryos is not formed, as cells of inner integument layers do not have callose deposits.

At the stage of the embryo organogenesis and active growth, a cuticle appears on the surface of the endosperm. This indicates that the growing young sporophyte isolates itself from the maternal tissues, and its further growth proceeds mainly due to the resources of endosperm cells. Concomitantly, the structural organization of endothelium cells undergoes significant changes. These cells again acquire meristematic features and continue to proliferate in coordination with the growth of the embryo and the surrounding endosperm. The main function of the endothelium at this stage is, in our opinion, the formation of a moist protective sheath around the growing embryo.

### 3.5. PCD of the Integument of Tomato Ovules

In contrast with the PCD of integument in the ovules of many unitegmal plants, the programmed death of integumental cells and consequent formation of a lysis zone seems to be a unique developmental feature of tomato ovules and possibly in other members of *Solanaceae*. For instance, in most members of the *Asteraceae* family, cells of inner layers of integumental parenchyma form the zone of dying integumental cells. In these cells, mucilage is secreted through the plasma membrane and accumulates in the periplasmic space. This results in the separation of the protoplast from the cell wall, and excessive accumulation of mucilage in periplasmic space induces the shrinkage of the protoplast. Eventually, the mucilage fills in the space between the cell wall and shrinking and degenerating protoplast, and integumental cells are replaced with mucilage, which contributes to the unobstructed growth of the embryo [13,15,16,17,18]. On the contrary, our study showed that in tomato, the mucilage zone was localized distantly from the dying integumental cells, and was formed by the endothelium and the endosperm. The mucilage was not accumulated in the periplasmic space but extruded through the cell walls into the intercellular space. Thus, the PCD in the integument and the formation of the mucilage zone during tomato ovule development are two different but synchronous processes.

### 3.6. Formation of Pseudo-Embryo

Our study showed that the ovule development in transgenic tomato with an infertile phenotype also began with the activation of PCD in the integument cells, leading to the detachment of the endothelium from the integument. However, in the absence of the growing endosperm, the endothelium did not proceed towards the next stage of the differentiation, but maintained its meristematic activity and continued to proliferate. Ultimately this resulted in the formation of a special tissue, which imitates the developing embryo, a pseudo-embryo. As the pseudo-embryo grew, the size of the integument also increased, yet inner layers of integument continued to die. Spatial restrictions for the pseudo-embryo proliferation may stimulate the endoreduplication of DNA and the formation of large polyploid nuclei in pseudo-embryo cells. It is well-known that endoreduplication is directly connected with the elevation of synthetic activity and widespread in plants [19,38,39]. Somatic polyploidization is common for many cells at the terminal stages of differentiation [40], and these cells usually have large nuclei and never enter mitosis [39,41]. Therefore, the presence of polyploid nuclei in the pseudo-embryo cells at this stage of growth may indicate the exit of cells from proliferation.

Thus, the study of the anatomical structure of the ovules from the developing fruit of transgenic tomato with morphological anomalies showed that pseudo-embryos formed in most of them. We conclude that the formation of pseudo-embryo in unfertilized ovules of transgenic tomatoes initiates the development of ovaries and the growth of parthenocarpic fruit, as was shown for the ovules of parthenocarpic tomato fruit, induced by auxin [6,7].

## 4. Materials and Methods

### 4.1. Plant Material

Objects of investigation was the tomato plant *Solanum lycopersicum* L., line ‘YaLF’, produced by vegetative growth plants (five to seven generations) of transgenic tomato lines expressing the *ac* gene, encoding chitin-binding protein from *Amaranthus caudatus* L. Tomato line YaLF was created from the cultivar Yamal, and used as a parental line to obtain the F1 Junior hybrid.

Transgenic plants were produced by *Agrobacterium*-mediated transformation using binary vector pBI121ac, containing targeting the *ac* gene under the cauliflower mosaic virus 35S RNA promoter (*CaMV35S*), the terminator of nopaline synthase (*NOS*) gene, and the *nptII* gene for the selection of transgenic plants. These transgenic plants were more resistant to *Phytophthora infestans* [9,42]. Wild type and transgenic plants were grown in plastic pots filled with soil, at standard agricultural conditions (22–25 °C day-time temperature and 18–19 °C night-time temperature, humidity 60–70%, illumination 2500 lx). Transgenic tomato plants were propagated by the grafting of lateral shoots, formed on the adult plants. All vegetative generations were obtained in the same way.

### 4.2. Light Microscopy and Conventional Electron Microscopy

Ovaries and ovules at different stages of development were fixed for 24 h in 2.5% glutaraldehyde (Merck, Darmstadt, Germany) prepared on 0.1 M Sorensen’s phosphate buffer (pH 7.2) and containing 1.5% sucrose. Then samples were washed, post-fixed in 1% OsO4 (Sigma-Aldrich, St. Louis, Missouri, USA), dehydrated in ethanol of increasing concentrations (30, 50, 70, 96, and 100%), propylene oxide and embedded in a mixture of Epon-812 and Araldite (Merck, Darmstadt, Germany), according to the standard protocol.

For light microscopy, semi-thin sections (1–2 μm) were prepared using glass knives and an ultramicrotome LKB-V (LKB, Sweden), placed on glass slides, stained with 0.1% methylene blue (Merck, Darmstadt, Germany), and embedded in epoxide resin. Samples were analyzed and photographed using an Olympus BX51 microscope (Olympus, Shinjuku, Tokyo, Japan) supplied with a Color View II camera (Soft Imaging System, Münster, Germany).

For electron microscopy, samples were sectioned with a diamond knife using an ultramicrotome LKB-V (LKB, Sweden), placed on formvar-coated grids, and stained with uranyl acetate and lead citrate. Then thin sections were analyzed and photographed using an electron microscope H-500 (Hitachi, Ibaraki, Japan) and JEM-1400 (Jeol, Akishima, Tokyo, Japan).

### 4.3. Staining of Cell Walls and Nuclei

The ovules were fixed for 2 h in 4% paraformaldehyde (Merck, Darmstadt, Germany) dissolved in 0.1 M phosphate-buffered saline (PBS). Then samples were washed in PBS, dehydrated in ethanol, and embedded in LR White (Merck, Darmstadt, Germany) according to the standard protocol [43]. Semi-thin sections (1–2 μm) were prepared using ultramicrotome LKB-V, mounted on glass slides, and incubated in a 0.1% solution of propidium iodide (Merck, Darmstadt, Germany) for 3 min. Samples were embedded in Moviol (Polysciences Inc., Eppelheim, Germany) and analyzed using the Olympus BX51 microscope (Olympus, Shinjuku, Tokyo, Japan) with standard filter sets for epifluorescence and equipped with digital camera Color View II FW (Germany).

### 4.4. Cytophotometry and Statistical Analysis

For evaluation of the ploidy level of pseudo-embryo cells, the ovules from green parthenocarpic fruits were isolated, incised with the razor blade, and fixed in ethanol/acetic acid mixture (3:1) for 3 h. Then the ovules were treated with 5N HCl for 40 min at 22 °C and stained with Schiff reagent (Merck, Darmstadt, Germany), according to the standard procedure. Stained cells of pseudo-embryo were visualized under the microscope, separated from other tissues, and used for evaluation of the DNA content using a SMP-20 cytophotometer Opton (Carl Zeiss, Obercochen, Germany) and 0.08–2.5 mm probes, and registered as arbitrary units. We examined three samples containing more than 200 nuclei; each sample was prepared from four to five ovules. The data were analyzed using the program Statistica 5.0, Student’s parametric criteria, and standard Microsoft Excel software packages.

### 4.5. Staining of Callose

The ovules were fixed for 1.5 h in 4% paraformaldehyde (Merck, Darmstadt, Germany) dissolved in 0.1 M PBS, washed in PBS, and embedded in 30% sucrose and polyvinylpyrrolidone (PVP40). Semi-thin sections were prepared as described earlier, mounted on glass slides, stained for 10 min in a water solution of anyline blue according to [36], and embedded in Moviol (Polysciences Inc, Eppelheim, Germany). Fluorescence of callose was registered using an Olympus BX51 microscope (Olympus, Shinjuku, Tokyo, Japan) with standard filter sets for epifluorescence, and equipped with digital camera Color View II FW (Germany).

## 5. Conclusions

Our study provides new and previously unknown information about the unique features of tomato ovule development. We found that the detachment of the endothelium from the integument occurs due to PCD of the integumental parenchyma cells, adjacent to the endothelium. The disconnection of the endothelium from the endosperm was associated with the transformation of the endothelium and peripheral layer of the endosperm into secretory cells. This is followed by the formation of the mucilage zone between both tissues. The accumulation of mucilage ensures independent and smooth growth of the endothelium alongside the endosperm.

We demonstrated that the differentiation of the integument in the fertilized tomato ovule is induced by the growth of the new sporophyte (embryo and endosperm) and leads to PCD. The development of the unfertilized ovule may be activated by pollination (or hormonal stimulation), which also triggers PCD in the integument. This process also activates the detachment of the endothelium, followed by its further proliferation and the consequent formation of the pseudo-embryo.

The final stages of the integument development depend on the presence of secreted lytic enzymes. In normally developing (fertilized) tomato ovules, the lytic enzymes are synthesized by the endothelium cells and used for the lysis of integument cells, undergoing the PCD. The lysis zone provides the space for the growth of the new sporophyte. In abnormally developing (unfertilized) ovules, the endothelium does not function as secretory tissue and forms the pseudo-embryo. Therefore, dying integumental cells that are not lyzed, accumulate and restrain the growth of the pseudo-embryo and the ovule. Ultimately, pseudo-embryo cells undergo PCD, while in normally developing (fertilized) ovules, the endothelium functions up to the full maturation of the seed.

Thus, our results first demonstrate distinctive structural and functional characteristics of the endothelium differentiation and its transformation into the pseudo-embryo during tomato ovule development. Our data may be helpful for studies on the expression of genes involved in endothelium differentiation, embryo development, as well as the development of new cellular and genetic tools in agrobiology and biotechnology for studying parthenocarpy, polyploidization and plant reproduction. Moreover, the developing tomato ovule appears to be a convenient and accessible model for studying the processes of terminal differentiation and PCD.

## Abbreviations

ER	Endoplasmic reticulum
PCD	Programmed cell death
PBS	Phosphate buffer saline
